# Mapping the Impact of Janus Kinase Inhibitors at a Cellular Resolution in Inflammatory Bowel Disease

**DOI:** 10.1016/j.jcmgh.2026.101867

**Published:** 2026-08-31

**Authors:** Jonas Louwers, Evelien Floor, Femke van Wijk, Yvonne Vercoulen, Bas Oldenburg

**Affiliations:** 1 Department of Gastroenterology and Hepatology, University Medical Center Utrecht, Utrecht, The Netherlands; 2 Center for Translational Immunology, University Medical Center Utrecht, Utrecht, The Netherlands; 3 Center for Molecular Medicine, University Medical Center Utrecht, Utrecht, The Netherlands

**Keywords:** Inflammatory Bowel Disease, Mucosal Immunology, JAK-STAT Signaling, Intestinal Epithelial Cells, Cytokine Signaling, Monocytes, Macrophages, Tofacitinib, T Cells

## Abstract

The advent of biologics and targeted oral small molecules has transformed the management of patients with inflammatory bowel disease (IBD), leading to increased rates of mucosal healing, fewer hospital admissions, and reduced need for surgery. In contrast to biologics that specifically target a receptor or its substrate, Janus kinase inhibitors (JAKis) interfere with several shared signaling pathways and therefore exert broader effects. Janus kinase–signal transducer and activator of transcription signaling (JAK-STAT) occupies a central position in multiple pro-inflammatory pathways and plays a pivotal role in the pathophysiology of autoimmune and inflammatory diseases. However, the pleiotropic effects of JAKis have greatly complicated the identification of cellular mediators of beneficial, adverse, or off-target effects, and this remains a challenge to date. This review summarizes current knowledge on the mechanisms of action of JAKis for the treatment of IBD, outlined by major cell types implicated in the pathophysiology of the disease. A deeper understanding of their effects on the diverse mucosal cell types is crucial to elucidate therapeutic mechanisms and explain treatment nonresponse.

**Summary**

This review provides a comprehensive overview of current knowledge regarding the effects of JAK inhibitors on cell types relevant to IBD, with a focus on human studies.

Inflammatory conditions such as inflammatory bowel disease (IBD), comprising ulcerative colitis (UC) and Crohn’s disease (CD), are characterized by excessive secretion of cytokines by immune and epithelial cells. For more than 60 types of cytokines and growth hormones, signals are transmitted via the Janus kinase–signal transducer and activator of transcription (JAK-STAT) pathway.^1^ There are 4 different JAK proteins (JAK1, JAK2, JAK3, and TYK2) that bind to cytokine and growth hormone receptors and function in variable pairs. Upon cytokine binding, the receptor tails dimerize, causing JAK proteins to become transphosphorylated. The activated JAKs subsequently phosphorylate the receptor tails, which then serve as docking sites for downstream STAT proteins (STAT1-6). Once bound, STAT proteins get phosphorylated and dissociate from the cytokine receptor. In the cytosol, the STATs form heterodimers or homodimers and translocate to the nucleus to initiate the transcription of cytokine-responsive genes. The effectiveness of new therapies targeting the JAK-STAT pathway underpins its central role in inflammation. To date, the following 3 JAK inhibitors (JAKis) have been approved for the treatment of IBD: tofacitinib, a pan-JAKi, and upadacitinib and filgotinib, both considered JAK1-selective inhibitors. Although all 3 JAKis have demonstrated efficacy in the treatment of UC, only upadacitinib has been approved for the treatment of CD.^2^, ^3^, ^4^, ^5^, ^6^ Of note, while filgotinib did not reach the co-primary end points at week 10, their attainment at week 58 suggests a delayed yet meaningful clinical benefit in patients with CD.^7^ This variability in efficacy across indications within the JAKis class raises questions regarding the underlying mechanisms contributing to this observation. While all JAKis block JAK-STAT signaling, their effects on different cell types remain elusive. In this review, we aim to provide an overview of the current literature on the effects of JAK inhibition at the cellular level. We performed a systematic literature search to identify studies reporting in vivo, ex vivo, or in vitro effects of JAKis on human intestinal cells, including cell lines, organoids, and co-cultures. When articles presented blood-based data alongside intestinal tissue findings, both were included. Studies exclusively reporting blood-based assays were excluded, as the gut represents the primary site of inflammation in IBD. Murine data presented within the included articles were also reported. Although all intestinal cell types were evaluated, we present only those with a substantial evidence base: monocytes, macrophages, T cells, and epithelial cells. This tissue-focused approach ensures that the review addresses intestinal mechanisms most relevant to IBD pathophysiology.

## Myeloid Cells

Myeloid cells form the principal cellular interface between innate and adaptive immunity. Therefore, delineating how JAKis modulate specific myeloid subsets may be crucial for elucidating the mechanisms that govern treatment response across different cell types. Both monocytes and macrophages play a pivotal role in the pathogenesis of IBD; monocytes are primarily found in the peripheral blood and can migrate to the gut, where they differentiate into monocyte-derived macrophages (MDMs), which are often short-lived and can perpetuate inflammation by the release of pro-inflammatory cytokines.^8^ Conversely, tissue-resident macrophages develop during embryogenesis and have self-renewing capacity independent of monocyte recruitment.^9^ Gut macrophages can adopt a pro-inflammatory phenotype, thereby contributing to the pathogenesis of IBD.^8^ Despite their physiological relevance, studies investigating intestinal tissue-derived monocytes and macrophages in IBD remain scarce. Peripheral blood-derived monocytes and MDMs, however, offer indirect yet informative insights into how JAKis affect myeloid cell function in IBD.

### Monocytes

JAKis suppress JAK-STAT pathway activation in vitro, as tofacitinib inhibited the phosphorylation of STAT1 (pSTAT1), STAT5, and STAT6 in cytokine-primed primary monocytes and suppressed interferon (IFN)-γ-induced pSTAT1 in primary CD14^+^ monocytes.^10^^,^^11^ Moreover, tofacitinib treatment abrogated *Campylobacter*-induced STAT3 phosphorylation in Tohoku hospital pediatrics-1 (THP-1) monocytes.^12^ These findings demonstrate that monocytes respond to JAK inhibition and suggest that monocyte-mediated mechanisms may contribute to the therapeutic effects of JAKis in IBD. However, circulating monocytes do not fully reflect the functional state of tissue-resident cells in the inflamed intestinal mucosa. Arribas-Rodríguez et al.^13^ demonstrated that ex vivo tofacitinib treatment suppressed JAK1 and JAK3 in lamina propria monocytes from patients with UC. Notably, this effect was disease-state dependent, with a selective reduction of JAK1 expression in active UC and decreases in both JAK1 and JAK3 during quiescent disease, suggesting that the inflammatory milieu may alter JAK expression and thereby influence JAKi sensitivity in tissue-resident monocytes. Importantly, reduced JAK-STAT phosphorylation does not necessarily translate into functional changes or shifts in monocyte abundance, underscoring the need for downstream validation. Transcriptomic analysis of intestinal biopsies from patients with CD treated with upadacitinib showed reduced expression of inflammatory monocyte-related genes in colon and ileum biopsies.^14^ Consistent with these findings, fewer monocytes were detected in the colon and blood of tofacitinib-treated mice after *Campylobacter* infection.^12^ Similarly, the abundance of inflammatory monocytes in the colon of mice with innate immune colitis decreased after treatment with ruxolitinib,^15^ a JAKi currently not approved for the treatment of IBD. Thus, JAK inhibition affects JAK-STAT signaling in monocytes and directly affects the abundance of monocytes in the gut.

#### Healthy and IBD monocytes show contrasting cytokine and activation responses to tofacitinib

The effect of tofacitinib on cytokine secretion by monocytes has been studied using peripheral blood monocytes from both healthy controls (HCs) and patients with IBD, often using granulocyte-macrophage colony–stimulating factor (GM-CSF) and IFN-γ co-stimulation to induce monocyte overactivation in vitro.^16^^,^^17^ Within this framework, tofacitinib reduced GM-CSF-induced tumor necrosis factor (TNF) α, interleukin (IL-6), and IL-10 secretion in HC monocytes.^10^ When IFN-γ was added, creating a more pronounced activation state, tofacitinib still reduced TNF-α and IL-6 secretion but no longer affected IL-10 (Figure 1).^10^ However, this inhibitory effect is stimulus-dependent. In monocytes stimulated with IFN-γ and lipopolysaccharide (LPS), tofacitinib had no significant effect on TNF-α, IL-6, or IL-10 secretion, although CXCL10 secretion was reduced,^23^ indicating a selective effect on immune cell recruitment. Other studies reported broader suppression, including reduced CXCL10, TNF-α, and IL-6, alongside increased IL-10 levels.^20^ Similarly, in *Campylobacter*-infected THP-1 monocytes, tofacitinib reduced the expression of CCL2, IL-6, and IL-12.^12^ These findings highlight that the effects of tofacitinib on the secretion of monocyte cytokines depend on stimulus combinations and are highly context-dependent. Whether these results translate to chronic inflammation in vivo remains unclear.

**Figure 1:**
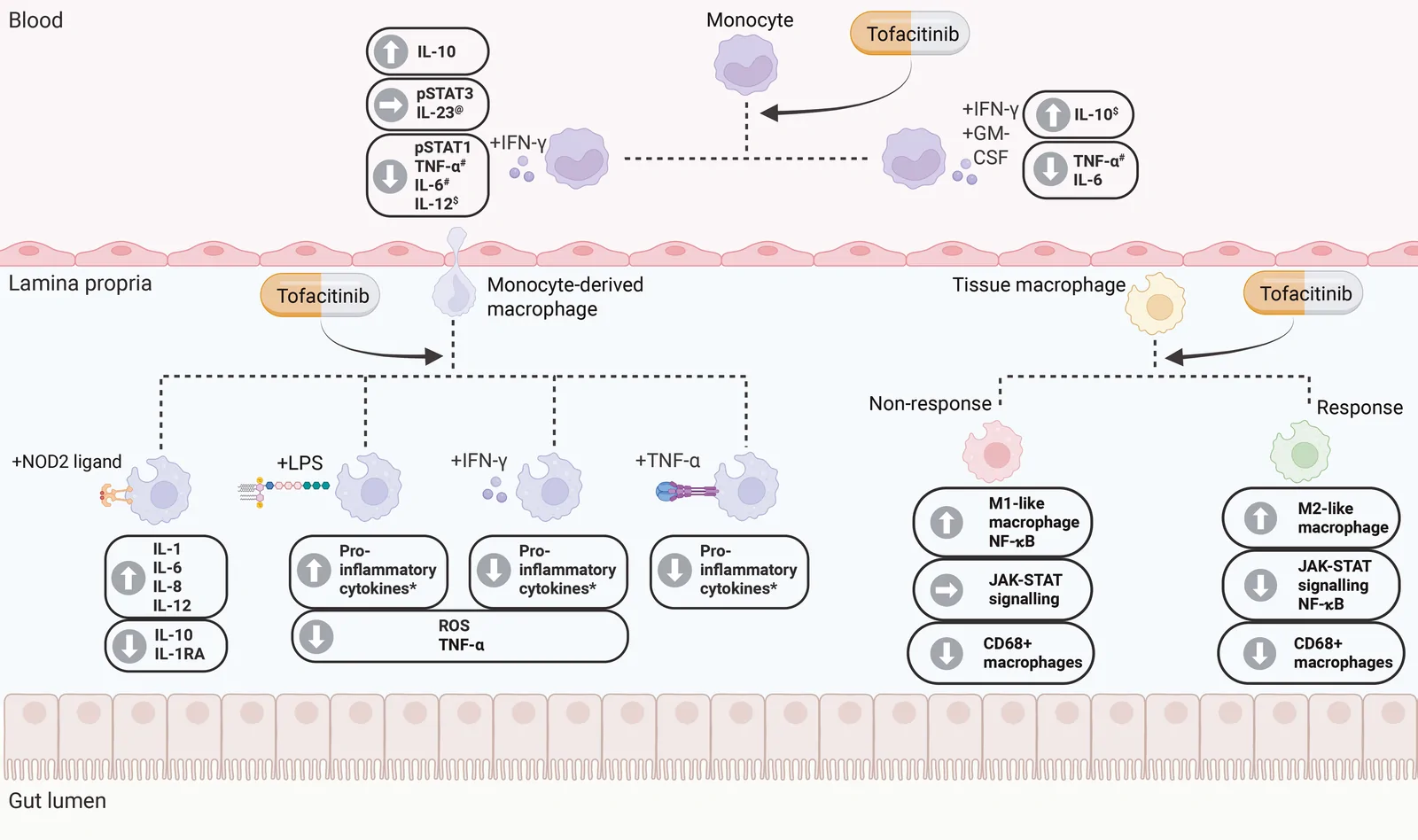
**Effects of tofacitinib on myeloid cells.** Summary of data on tofacitinib’s effects on monocytes, monocyte-derived macrophages, and tissue-resident macrophages. Cell location reflects anatomical compartment; ex vivo co-stimulatory conditions are indicated where applicable. *Arrows* indicate direction of effect: upward (increase), downward (decrease), horizontal (no change). Tofacitinib treatment of IFN-γ-stimulated monocytes results in decreased STAT1 phosphorylation together with lower levels of TNF-α (not in CD), IL-6 (not in CD), and IL-12 (not in HCs) and increased IL-10 levels.^11^ Tofacitinib does not affect STAT3 phosphorylation or IL-23 levels (in HCs and CD).^11^ Stimulation with GM-CSF/IFN-γ increases IL-10 levels (not in HCs) and inhibits the release of TNF-α (not in CD) and IL-6.^10^ In MDMs, tofacitinib induces pro-inflammatory responses stimulated with LPS or NOD2 ligands,^18^^,^^19^ but not with IFN-γ or TNF-α.^19^ Under combined LPS and IFN-γ stimulation, tofacitinib attenuated M1-associated inflammatory outputs.^20^^,^^21^ Tissue macrophages of nonresponders show a more M1-like phenotype with increased NF-κB signaling and unaltered JAK-STAT signaling upon tofacitinib treatment, whereas macrophages of responders show a more M2-like phenotype with decreased NF-κB and JAK-STAT signaling.^19^ In both responders and nonresponders, a decrease in CD68^+^ macrophages was observed.^22^ ∗Pro-inflammatory cytokines: CXCL1, CXCL10, IL-1β, IL-23A, IL-6, INHBA, CLEC5A. ^@^Only in HC and CD, decreased in UC. ^$^Only in UC and CD, unaltered in HC. ^#^Only in HC and UC, unaltered in CD.

Beyond the effects observed in HC monocytes, tofacitinib also modulates cytokine secretion in monocytes from patients with IBD with active disease, although its impact differs between UC and CD. In UC monocytes, tofacitinib inhibited the release of TNF-α and IL-6 following IFN-γ or GM-CSF/IFN-γ stimulation, whereas this inhibitory effect was not observed in CD monocytes following IFN-γ stimulation.^10^^,^^11^ Strikingly, at lower tofacitinib concentrations, the release of IL-6 and TNF-α was significantly increased in UC monocytes, whereas IL-23 release was significantly increased in both UC and CD.^11^ This suggests that low-dose treatment could counterintuitively worsen inflammation in IBD. However, at a high tofacitinib concentration, IL-23 significantly decreased in UC but not in CD^11^ (Figure 1). Stimulation with tofacitinib and IFN-γ or GM-CSF/IFN-γ increased the release of IL-10 in the monocytes of patients with UC and CD with active disease, although this increase was less pronounced than that in HCs.^10^^,^^11^ This divergent response suggests that inflammatory reprogramming of IBD monocytes partially constrains the JAK-dependent IL-10 induction that marks tofacitinib-driven anti-inflammatory polarization. Consistent with this, high-dose tofacitinib inhibited IFN-γ-induced release of IL-12 in both UC and CD monocytes. Notably, a stronger inhibitory effect was observed in UC, which may help explain the efficacy of tofacitinib in UC but not in CD.^11^ These inhibitory effects were absent in HC monocytes, suggesting that epigenetic differences may underlie the variable responses to JAK inhibition.^24^ These findings underscore the importance of using physiologically relevant in vitro models that reflect the complex in vivo environment.

Monocytes express various regulatory membrane receptors that play a role in inflammation. Their expression can be assessed to characterize monocyte function, such as CD80 and CD86, which are co-stimulatory receptors required for T cell activation, and CD206, a mannose receptor acquired upon maturation into MDMs.^25^ CD80 and CD86 are upregulated on the monocyte membrane upon pro-inflammatory activation. CD39, on the other hand, is an ectonucleotidase expressed on the membrane associated with an anti-inflammatory monocyte phenotype since it breaks down pro-inflammatory ATP into AMP.^26^ JAKis modulate the expression of these important immune regulatory molecules with diverging effects in HC, UC, and CD monocytes. For example, upadacitinib and tofacitinib inhibited the expression of CD86 in IFN-α-stimulated CD14^+^ monocytes from HCs with different efficacies (100% and 50% inhibition, respectively).^27^ Inhibition was similar for both JAKis when monocytes were stimulated with IFN-γ. Besides, treatment of healthy IFN-γ- or GM-CSF/IFN-γ-stimulated monocytes with tofacitinib resulted in the downregulation of CD39, CD80, and CD206.^10^^,^^11^ In GM-CSF/IFN-γ-stimulated UC monocytes, tofacitinib only downregulated CD39, whereas in CD monocytes, CD80 was also inhibited.^10^ However, in IFN-γ-stimulated monocytes, CD80 was downregulated in both UC and CD, whereas CD206 was only inhibited in UC.^11^ Notably, CD39 was significantly more downregulated in IFN-γ-stimulated UC monocytes compared with CD monocytes, potentially promoting inflammation.^11^ This again shows that monocytes from patients with UC and CD respond differently to JAK inhibition than those of HCs. Overall, JAK inhibition appears to shape monocyte phenotype according to both the inflammatory stimulus and the underlying disease state, with downstream effects that may either attenuate or promote inflammation.

### Monocyte-Derived Macrophages

Most mechanistic insights into JAK-STAT pathway inhibition in macrophages are derived from in vitro differentiation models such as MDMs, bone marrow-derived macrophages, and induced pluripotent stem cell (iPSC)-derived macrophages, reflecting the paucity of studies directly examining intestinal macrophages. Despite being derivative models, their use is biologically grounded. Classical monocytes are recruited to the gut via CCR2-CCL2 signaling and, during inflammation, predominantly differentiate into pro-inflammatory CD14^hi^HLA-DR^low^CD163^low^CD64^+^ macrophages that secrete a broad array of cytokines capable of perpetuating inflammation.^8^ However, since these models omit the native intestinal microenvironment, findings from such systems should be interpreted with caution.

#### Differentiation stage dictates timing-dependent macrophage reprogramming

Tofacitinib’s effects on macrophage polarization appear to be critically influenced by the timing of JAK inhibition. When CD14^+^ monocytes were differentiated with M-CSF into MDMs and exposed to M0 (M-CSF), M1 (LPS/IFN-γ), or M2 (IL-4) polarizing conditions, the presence of tofacitinib during both differentiation and polarization suppressed M1-associated mRNA transcripts (*TNF* and *NOS2*) and increased *IL10* across all conditions.^20^ Under M0/M2 conditions, tofacitinib induced M2-associated markers (*ARG1* and *IL10*). However, when macrophages were differentiated without tofacitinib and only later restimulated in its presence, M1 suppression persisted, but M2-promoting features disappeared, indicating that tofacitinib influences differentiation programming more than committed phenotypes.

De Vries et al. showed that tofacitinib and JAK1-selective (but not JAK3-selective) inhibition suppress IFN-γ-linked signaling, reducing STAT1 phosphorylation and IFN-γ-induced transcripts.^23^ This produced partial M2 marker shifts (increased CD206/CD64 ratio in mice and CD163/CD64 in human induced macrophages) and increased the production of IL-10, indicating the acquisition of regulatory features without full IL-4-driven M2 conversion. Notably, this increase depends on the comparator; IL-10 may rise relative to unstimulated baseline but may still be reduced relative to the strong IL-10 induction seen with IFN-γ + LPS stimulation. Under M2 polarizing conditions, JAK1i/tofacitinib did not further increase IL-10 or CD206, suggesting either a ceiling effect or a limited ability of JAK inhibition to augment an established IL-4-driven phenotype.

Lethen et al. similarly showed these polarization state–dependent effects of tofacitinib by treating M-CSF-derived MDMs from HCs and from patients with UC or CD with tofacitinib.^28^ Under M1-like conditions, tofacitinib strongly increased the anti-inflammatory cytokine IL-10 and reduced the immunostimulatory/co-stimulatory surface molecules CD80, CD86, CD83, and CD40, which are associated with M1-like activation, whereas the effects on pro-inflammatory cytokines (TNF-α, IL-1β, IL-6, IL-12, and IL-23) were limited or mixed. Under IL-4-driven M2-like conditions, tofacitinib contrastingly reduced IL-10 and the M2-associated marker CD206, consistent with impaired M2-like differentiation. These patterns were broadly similar across HCs, UC, and CD, arguing against a major disease-specific effect in this in vitro setting. This is notable because circulating monocytes are known to be cytokine conditioned before entering tissues, even in quiescent IBD.^29^ The broadly comparable ex vivo response to JAK inhibition suggests that the polarization state may be a more important determinant of tofacitinib response than the disease label alone. In summary, JAK inhibition via JAK1-linked IFN-γ-STAT1 dampening reduces inflammatory activation and co-stimulatory capacity of monocytes and MDMs while permitting IL-10 production, with effects dependent on timing, JAKi dose, and donor context.

#### Paradoxical immunologic effects of JAK inhibition in the gut

Hedl et al. examined how JAK inhibition reshapes human myeloid cell responses under conditions meant to mimic microbial sensing in the gut.^18^ They demonstrate that strong reduction of JAK-STAT pathway function uncouples pro- and anti-inflammatory outputs; acute pattern recognition receptor (PRR) stimulation markedly increased pro-inflammatory cytokines (IL-6 and IL-12) while decreasing the levels of anti-inflammatory cytokines (IL-10 and IL-1RA). This occurs because PRR stimulation normally induces an autocrine/paracrine anti-inflammatory brake, notably IL-10, IL-4, IL-22, and thymic stromal lymphopoietin, which suppresses pro-inflammatory cytokines and depends on intact JAK signaling for optimal function. Inhibiting the pathway, therefore, weakens this feedback and tips responses toward inflammation. Most importantly, for IBD relevance, Hedl et al. extended their findings beyond MDMs to primary human intestinal myeloid cells isolated from colonic specimens (reported CD11c purity >75%). In these tissue-derived cells, multiple JAKis, including tofacitinib, caused dose-dependent increases in pro-inflammatory cytokines (IL-1β and IL-8) following exposure to live *Salmonella typhimurium*. Hedl et al. emphasize that paradoxical pro-inflammatory effects emerge when JAK inhibition pushes signaling below a functional threshold. Anti-inflammatory cytokines decrease progressively as JAK signaling drops, whereas pro-inflammatory cytokines shift from an initial decrease to an increase under stronger inhibition.

Melón-Ardanaz et al. similarly report that the effects of tofacitinib on myeloid cells depend on the inflammatory context; in MDMs, tofacitinib hyperactivated responses to LPS but not TNF-α or IFN-γ (Figure 1).^19^ Mechanistically, this paradox required the inhibition of IL-10 signaling, consistent with IL-10 being strongly induced by LPS but not by TNF-α or IFN-γ. By contrast, fibroblasts, which do not produce IL-10 under these stimuli, did not show tofacitinib-driven hyperactivation.^19^ Consistent with this context dependency, a microbiota-conditioned epithelial-macrophage co-culture experiment found that tofacitinib significantly downregulated the chemokines CCL2 and CXCL10 after LPS challenge, while leaving most of the other inflammatory outputs unchanged.^30^ Notably, IL-10 was not affected, which supported a selective rather than global suppression profile in this setting.

### Tissue Macrophages

Emerging evidence indicates that the intestinal immune landscape substantially differs between the colon and the ileum, with tissue location accounting for a greater proportion of transcriptomic variance in lamina propria phagocytes than the inflammation status or disease type.^31^ Although tofacitinib and ruxolitinib suppress LPS-induced TNF-α and IL-8 production more strongly in ileal than in colonic CD11b^+^ cells, the specific phagocyte subsets driving this differential response remain unclear.^31^ Direct evidence of the effects of JAKis on tissue macrophages in IBD remains limited. In CD, only upadacitinib has been studied. Aguilar et al.^14^ reported reduced expression of macrophage-associated gene signature in responders, particularly in colonic tissue, whereas Wang et al.^32^ found no change in macrophage abundance in the same dataset.^14^^,^^32^ These findings suggest functional reprogramming of resident macrophages rather than reduced recruitment. This interpretation, however, should be viewed with caution, considering the limitations of bulk RNA sequencing, which lacks the resolution to distinguish monocytes and macrophages reliably and has quantitative limitations in estimating cell abundance estimates.

In the context of UC, tofacitinib altered macrophage-associated chemokines in stimulated biopsy explants, specifically decreasing monocyte chemoattractant protein 1 (MCP-1/CCL2) and increasing macrophage-derived chemokine (MDC/CCL22).^33^ However, the specific contribution of macrophages was inferred rather than directly measured. Flow cytometry analyses on isolated macrophages from HC, quiescent UC, and active UC tissues showed that tofacitinib had minimal direct effects. Only macrophages from active UC showed reduced JAK1 activation, while other functional markers (IL-1β, IL-6, IL-10, and TLR2) and macrophage cell numbers were unchanged.^13^ The absence of macrophage subtyping restricted the detection of subtle phenotypic differences, while the ex vivo use of tofacitinib reduced the physiological relevance of the findings.

Both limitations were mitigated by single-cell RNA sequencing of posttreatment biopsies from patients with UC receiving tofacitinib. After therapy, responders had fewer inflammation-associated inhibin subunit beta A^+^ macrophages and a more pronounced homeostatic M2 phenotype of folate receptor 2^+^ macrophages compared with those in nonresponders (Figure 1).^19^ Before the administration of tofacitinib, IL-10 signaling was higher in macrophages and plasmablasts from nonresponders. These in vivo data underline a potential paradox of JAK inhibition: by simultaneously blocking both pro-inflammatory and suppressive cytokine signaling, tofacitinib may inadvertently disrupt a critical anti-inflammatory brake, promoting net pro-inflammatory activity in these cells. In nonresponders, who appear to depend more heavily on IL-10-mediated suppression, this loss of compensatory signaling could shift the myeloid cell phenotype toward net pro-inflammatory activity, particularly if JAK-dependent inflammatory pathways remain insufficiently inhibited. This effect may be exacerbated in the inflamed gut, where microbial triggers such as LPS and nucleotide-binding oligomerization domain-containing protein 2 (NOD2) ligands promote sustained IL-10 activity, rendering myeloid cells sensitive to disruption of this pathway. This interpretation is supported by the observation that, although van Gennep et al. found a decline in mucosal CD68^+^ macrophages after tofacitinib administration in both responders and nonresponders, reductions in mucosal T cell markers were more response restricted,^22^ suggesting that macrophage reduction alone is insufficient to define therapeutic success.

## T Lymphocytes

T cells are important drivers of inflammation in IBD, as demonstrated by the therapeutic efficacy of biologicals targeting the IL-23/T helper 17 cell (Th17) axis and T cell gut homing.^34^ Within T cells, marked functional variability based on contextual cues exists, many of which are dictated by their locational context.^35^^,^^36^ Here, we provide an overview of studies describing the effects of JAKis on T cell populations across distinct anatomical compartments—specifically, blood and intestine—most of which concern tofacitinib. There remains a scarcity of evidence regarding JAKi effects on human T cells, particularly the gut resident subsets. While murine studies provide complementary insights, these findings should be interpreted with caution when extrapolating to humans.

### CD4^+^ T Lymphocytes

#### Tofacitinib differentially modulates Th subsets and trafficking receptors

Among naive CD4^+^ T cells from HCs cultured under Th1, Th2, or Th17 polarizing conditions, Th17 cells appeared most sensitive to tofacitinib. This conclusion was supported by complementary protein-, RNA-, and flow cytometry–based readouts, including reduced proliferation, lower IL-17, IL-17A, and IL-22 expression, and diminished retinoid acid receptor-related orphan receptor gamma t (RORγt) levels (Figure 2).^20^ Th1 cells exhibited decreased IFN-γ production and T-bet expression, although with less pronounced antiproliferative effects than observed in the Th17 subset. By contrast, Th2 cells appeared unaffected in terms of differentiation, proliferation, and cytokine production. However, this apparent unresponsiveness may partly reflect the inherently lower activation potential of Th2 cells.

**Figure 2:**
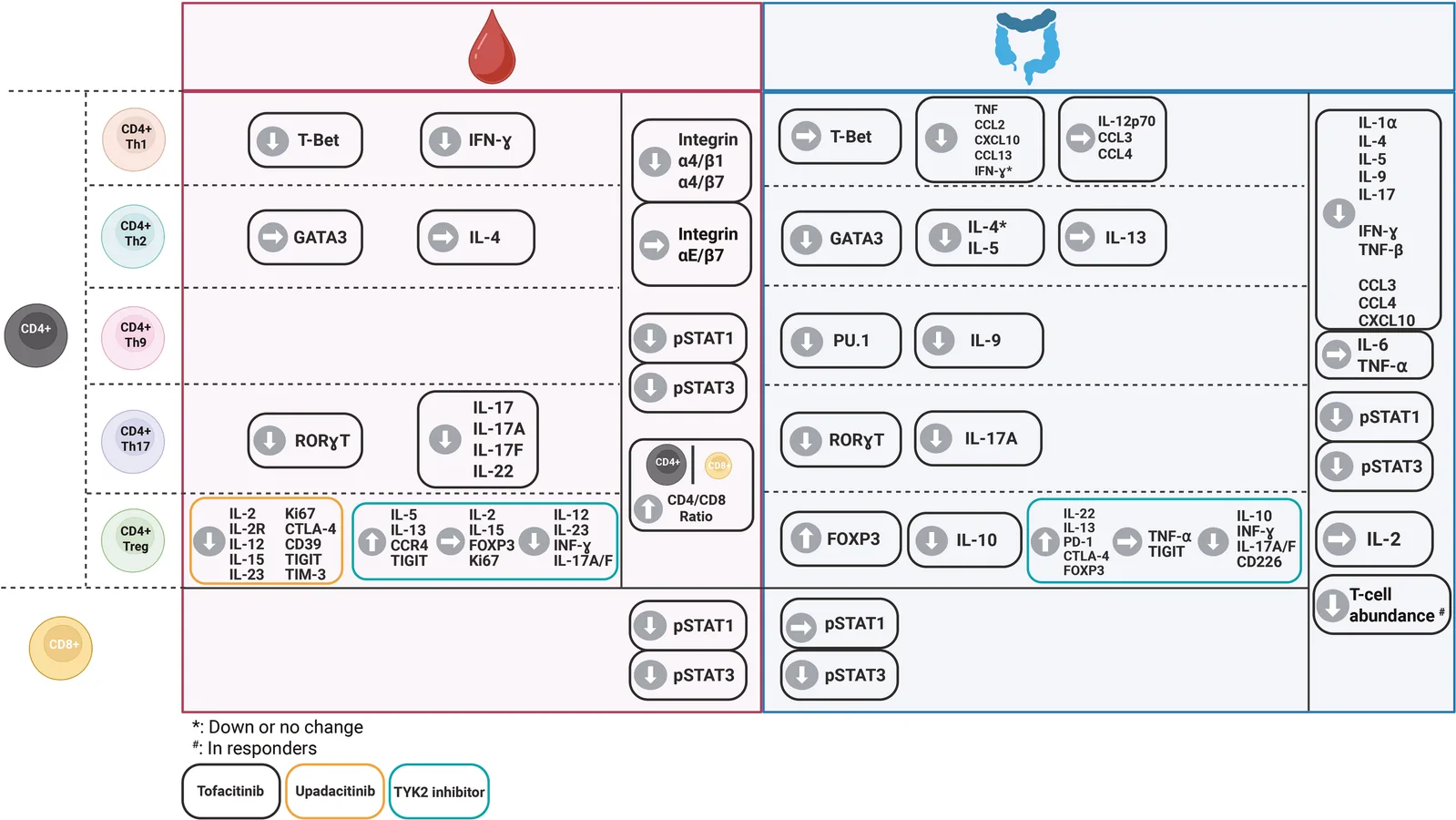
**Effects of JAK inhibition on human Tcell subsets.** Summary of currently available data on the effects of JAK inhibition on human T cells. The vertical organization reflects anatomical compartment (blood: red; gut: blue), whereas the horizontal arrangement distinguishes T cell subsets (CD4^+^, CD8^+^, and CD4^+^ Th subsets). *Arrows* indicate the direction of the reported effect: upward for increase, downward for decrease, and horizontal for no change. Properties with conflicting results are marked with an asterisk (∗). Tofacitinib demonstrates broad anti-inflammatory effects on T cells.^20^^,^^33^^,^^37^^,^^38^ Compartment-specific differences within T cell subsets are evident in the Th1 and Th2 populations, but such variation is absent in the Th17 subset. Although both upadacitinib and TYK2 inhibition suppress Th1/Th17 differentiation, selective TYK2 inhibition preserves (and can enhance) CD4^+^ Treg phenotype and suppressive function because it does not inhibit IL-2-driven JAK1/3-STAT5 signaling.^39^ Although direct data on CD8^+^ T cells are limited, the observed increase in the CD4/CD8 ratio suggests greater susceptibility of CD8^+^ cells to the effects of tofacitinib.^33^ Reduction in mucosal T cell abundance was associated with an endohistological response to tofacitinib.^22^

In a more clinically relevant model, using T cells from patients with active UC, Gerlach et al. observed a broad suppression of pro-inflammatory cytokines across Th1, Th2, Th9, and Th17 subsets due to tofacitinib.^37^ Contrary to the results presented by Texler et al., only Th2-related cytokines (IL-4 and IL-5) were significantly reduced when T cells from patients with inactive UC were exposed to tofacitinib. Interestingly, the same experiment shows a sharp decrease in α4/β1 and α4/β7 integrin expression (Figure 2), thereby limiting the gut-homing potential of CD4^+^ T cells, while the unchanged expression of αE/β7 integrin could theoretically sustain gut retention capacity.^40^ Complementary tissue-level evidence is provided by a retrospective histologic analysis of colonic biopsies from patients with UC, in which pretreatment mucosal IL-17A^+^ mononuclear cell infiltration was significantly higher in tofacitinib nonresponders than in responders (38.2% vs 21.2%).^41^ This identifies the baseline Th17 mucosal burden as a candidate predictor of treatment failure, though the small single-center design and restriction to a single JAKi limit generalizability.

#### JAK inhibition attenuates gut T cell activation

Ex vivo tofacitinib treatment of biopsies from patients with active UC reduced Th2, Th9, and Th17 lineage-defining markers; left Th1 markers unchanged; and increased those of regulatory T cells (Tregs).^37^ The absence of significant changes in biopsies from patients with quiescent UC suggests that JAK inhibition predominantly modulates active inflammation. Brand et al. similarly observed a heterogeneous cytokine response in stimulated UC biopsies but with selective suppression of Th1-related cytokines, while Th2 and Th17 cytokines were largely unaffected.^33^ Although this methodology captures tissue-level responses, the mixed cellular composition prevents direct attribution of cytokine production to specific T cell subsets. The distinct pattern may also reflect differential effects of the stimulants used (IL-6, IL-22, and IFN-α) on CD4^+^ T cell subset activation.^33^ By contrast, Kaboub et al. described broader general anti-inflammatory effects of tofacitinib in lamina propria lymphocytes from both the colon and the ileum, as assessed by flow cytometry.^38^ The effect of tofacitinib on Tregs is generally stimulatory, as indicated by increased numbers of FOXP3^+^ cells. This finding is underpinned by murine data from the gut^37^^,^^42^ and from the spleen and mesenteric lymph nodes.^42^ Tuomela et al. add nuance by exploring the differential effects of JAK1/3 inhibition with upadacitinib vs a TYK2 inhibitor. Both compounds suppressed Th1 and Th17 differentiation, whereas only TYK2 inhibition preserved Treg function, consistent with its sparing of IL-2 signaling.^39^ This selective Treg-sparing effect was also observed in lamina propria mononuclear cells from patients with IBD, suggesting a theoretical advantage for TYK2 inhibition over upadacitinib. However, selective TYK2 inhibitors have thus far failed to surpass placebo in phase II trials for both UC and CD, leaving this inferred advantage without clinical validation.^43^

Investigating the transcriptional effects of upadacitinib in patients with CD, Aguilar et al. found a decrease in the Th1 lineage marker TBX21 in patients with colonic CD who responded to treatment.^14^ Notably, this decrease was not observed in patients responding to anti-TNF therapy, indicating a possible upadacitinib-specific effect on the Th1 subset of colonic T cells, as similar results were not found for genes related to other T cell subsets.

### CD8^+^ T Lymphocytes

Tofacitinib reduces the proliferation of peripheral blood CD4^+^ and CD8^+^ T cells in both HCs and patients with UC or CD, with a preferential effect on CD8^+^ T cells, reflected by an increased CD4^+^/CD8^+^ ratio and strong inhibition of naive CD8^+^ T cell proliferation.^20^ The only available human data on intestinal CD8^+^ T cells show that tofacitinib reduces pSTAT3 but not pSTAT1 (Figure 2) in colonic explants from patients with UC, indicating JAK-STAT pathway engagement without clarifying functional effects.^33^ This limited human intestinal evidence should be interpreted as indicating that CD8^+^ T cells remain underexplored as targets of JAK inhibition, rather than as implying limited biological relevance. In a murine colitis model, tofacitinib increased the intestinal CD4^+^/CD8^+^ ratio and reduced IFN-γ^+^ and IL-2^+^ CD8^+^ T cells in the lamina propria and epithelium, while effects in mesenteric lymph nodes and spleen were limited, suggesting a predominant impact at primary sites of inflammation and a broader systemic effect on T cell composition.^42^ Whether these findings generalize to the human intestine remains unclear, given fundamental differences between murine colitis models and human IBD pathophysiology. Taken together, despite discrepancies in the affected T cell subsets, the overall evidence indicates that JAK inhibition attenuates gut T cell inflammation and that this appears to contribute to therapeutic response, with recent mucosal data showing that reductions in T cell markers are preferentially observed in patients with UC achieving histoendoscopic improvement, whereas macrophage and neutrophil markers decline even in nonresponders.^22^

## Epithelium

The epithelium plays an essential role in maintaining intestinal homeostasis by not only acting as a physical barrier but also secreting mucus and mediating epithelial-immune interactions.^44^^,^^45^ Disruption of this barrier function may result in inflammation and the eventual development of IBD.^46^, ^47^, ^48^ The intestinal epithelium comprises various cell types, with enterocytes accounting for up to 80% of its cellular composition. Since most in vitro models are based on enterocytes, this cell type is most extensively studied in the setting of JAK inhibition. Tofacitinib inhibited IFN-γ-induced phosphorylation of JAK1 as well as downstream STAT1 and STAT3 in T84 human colonic adenocarcinoma cells.^49^ In addition, it inhibited IL-6-induced phosphorylation of JAK2 and STAT3 in HT-29 cells^50^ and STAT1/3 phosphorylation in a co-culture of THP-1 macrophages with Caco-2BBe cells.^51^ Moreover, the JAK1/2-specific inhibitor ruxolitinib inhibited TNF-α-induced phosphorylation of JAK1/2 and STAT3 in HCT116 colonic epithelial cells (Figure 3).^56^ Ex vivo stimulation of murine intestinal biopsies with IFN-λ in the presence of tofacitinib or filgotinib also abrogated STAT1 phosphorylation.^59^ Surprisingly, the in vivo effect of JAK inhibition on JAK-STAT activation was only studied in an oxazolone-induced colitis mouse model, where tofacitinib lowered the numbers of pSTAT3-positive epithelial cells.^37^ In these mice, suppressor of cytokine signaling 1 (SOCS1) was upregulated on tofacitinib treatment, whereas SOCS3 levels remained unchanged. SOCS proteins are negative regulators of the JAK-STAT pathway, and hence, increased levels of SOCS1 may indicate induction of negative feedback in colonocytes upon JAKi treatment. Finally, tofacitinib pretreatment of TNF+Poly(I:C)-stimulated colonoids from non-IBD and UC patients prevented STAT1 phosphorylation and suppressed the TNF+Poly(I:C)-induced upregulation of MHC-II in UC colonoids.^60^ Overall, there is strong evidence to support that the pan-JAKi tofacitinib indeed interferes with JAK-STAT activation not only in epithelial cell lines and UC colonoids but also in a mouse model of colitis. Still, studies using other JAKis are scarce or lacking, similar to studies addressing the in vivo effects on human enterocytes in IBD, particularly in CD.

**Figure 3:**
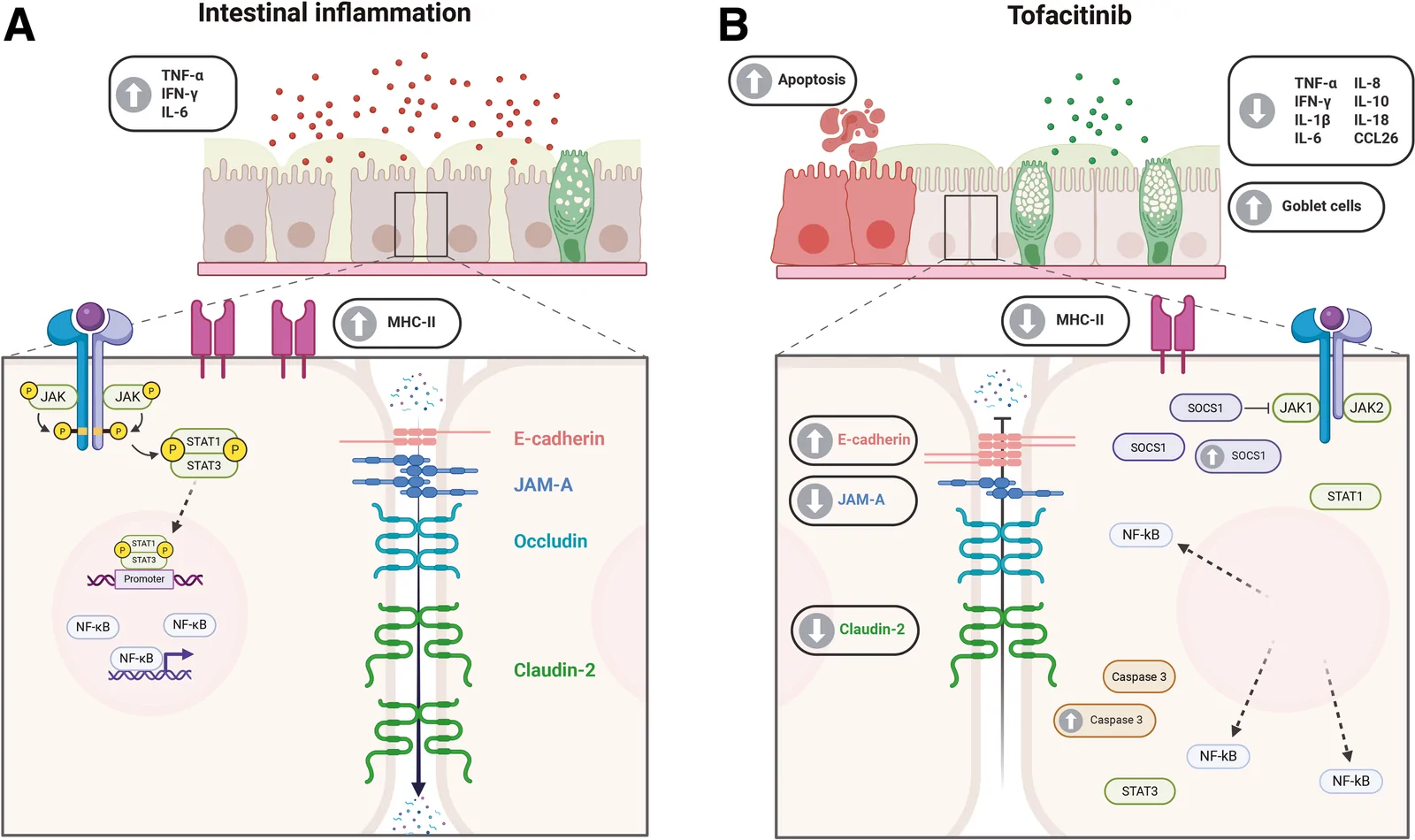
**Effect of tofacitinib on epithelial inflammation and barrier integrity.** An overview of the intestinal epithelium during inflammation and after JAK inhibition. *Arrows* indicate direction of effect: upward (increase), downward (decrease), horizontal (no change). (*A*) During intestinal inflammation, the release of pro-inflammatory cytokines by epithelial cells is increased, and goblet cells are depleted.^52^ This is accompanied by increased JAK-STAT signaling, increased MHC-II levels, and nuclear localization of NF-κB. Epithelial barrier integrity is compromised because of increased CLDN2 and decreased E-cadherin levels.^53^, ^54^, ^55^ (*B*) Following tofacitinib treatment, the release of pro-inflammatory cytokines is inhibited,^20^^,^^50^^,^^56^, ^57^, ^58^ and goblet cells are restored,^14^ accompanied by decreased JAK-STAT signaling^49^, ^50^, ^51^^,^^56^^,^^59^ and increased SOCS1 levels.^37^ NF-κB is translocated to the cytosol,^50^ and MHC-II levels are decreased.^60^ Caspase-3 levels are higher, resulting in increased cell death.^37^

#### Tofacitinib modulates junctional proteins to alter epithelial barrier integrity

IBD features a disrupted mucosal barrier comprising the epithelial layer and the overlying mucus layer. Hence, the effects of JAKis on epithelial barrier integrity have been extensively studied by measuring the permeability of small molecules and the transepithelial electrical resistance (TEER). In human colonic T84 monolayers, tofacitinib alone did not affect TEER, but IFN-γ also significantly reduced it, indicating barrier disruption. Co-treatment with tofacitinib fully restored the TEER and similarly reversed the IFN-γ-induced increased permeability of 4-kDa FITC-dextran (FD4).^49^ In the same study, the effect of adding tofacitinib either before or after IFN-γ treatment was assessed, and the results showed that posttreatment (reversion) significantly increased TEER but not as potently as in pretreatment (prevention), whereas tofacitinib was equally effective in both preventing and reversing FD4 permeability.^49^ Validation in human colonic organoids confirmed that tofacitinib prevented IFN-γ-induced FD4 flux and barrier loss.^49^ Treatment of organoid monolayers derived from HCs and from patients with UC and CD with tofacitinib significantly reversed cytokine-induced TEER loss and increased FD4 permeability.^61^ In addition, pretreatment of intestinal organoids derived from patients with CD with filgotinib significantly reduced FD4 permeability, further supporting a barrier-protective effect of JAK inhibition.^62^ By contrast, in TNF-α-treated Caco-2 monolayers, FD4 permeability could not be restored by tofacitinib.^63^ Interestingly, results from epithelial-macrophage co-culture systems are more heterogeneous. Co-culture of Caco-2BBe cells with THP-1 macrophages showed a significant increase in TEER with decreased FD4 permeability, both of which were normalized by tofacitinib.^51^ In contrast, other Caco-2/THP-1 models reported either decreased TEER upon macrophage stimulation in combination with tofacitinib^21^ or no significant TEER changes when macrophages were stimulated with LPS in the presence of tofacitinib.^30^ In a transverse colon/THP-1 co-culture, tofacitinib alone did not affect TEER; however, it restored the barrier function by reversing IFN-γ and LPS-induced TEER loss.^64^ These studies demonstrate that the effects of tofacitinib on epithelial barrier function are highly context dependent and vary according to the inflammatory stimulus and the specific in vitro model used.

Mechanistically, epithelial barrier regulation depends on the assembly and regulation of tight junction (TJ) proteins, including occludins (OCLN), claudins (CLDN), and zonula occludens (ZO) adapter proteins. Other proteins important for maintaining epithelial structural integrity are adherens junctions such as E-cadherin (CDH1) and cytoplasmic catenin cofactors.^53^ Tofacitinib treatment of HT-29 cells increased *CDH1* mRNA expression but had no effect on *OCLN* or *CLDN1*. In addition, it did not normalize E-cadherin protein levels in TNF-α-treated HT-29 cells or the colonic organoids of patients with UC.^63^ By contrast, preincubation of intestinal organoids derived from patients with CD with filgotinib partially restored E-cadherin levels, suggesting improved epithelial junctional integrity.^62^ Similarly, in dextran sodium sulfate (DSS)-induced colitis mice, tofacitinib normalized E-cadherin levels,^50^ further emphasizing that context matters, as DSS-induced colitis mimics acute epithelial damage in vivo. Additionally, the variable concentrations of tofacitinib used in these studies may contribute to discrepancies, underscoring the need for further, more comparable studies with human colonic tissues.

Treatment of TNF-α-stimulated HT-29 cells with tofacitinib did not restore increased *CLDN1* mRNA levels.^63^ However, in TNF-α-treated Caco-2 cells, CLDN1 levels increased upon tofacitinib treatment.^65^ CLDN1 belongs to the barrier-forming group of CLDN that are responsible for increasing barrier integrity, whereas the pore-forming CLDN increase permeability. CLDN2, a pore-forming CLDN, is reportedly increased in CD and UC.^54^^,^^55^ Treatment of T84 cells with IFN-γ increased CLDN2 protein levels, which can be partially normalized by treatment with tofacitinib.^49^ The CLDN2 promoter contains a STAT-binding motif, and upon IFN-γ stimulation, STAT1 induces CLDN2 transcription.^66^ Tofacitinib prevented CLDN2 promoter activity in HT-29 cells, while no significant changes in CLDN1, CLDN4, and CLDN15 were observed (Figure 3).^49^ Tofacitinib treatment did not affect ZO-1 or OCLN levels in T84 monolayers and human colonoids.^49^ Although there was no effect on ZO-1 protein levels, the number of ZO-1 intercellular gaps increased upon IFN-γ treatment of T84 cells, which disrupted the regular chicken wire pattern that ZO-1 proteins form to establish functional TJs.^49^ Tofacitinib both prevented and restored IFN-γ-induced disruptions, suggesting that increased CLDN2 levels during inflammation cause a mislocalization of ZO-1, resulting in decreased TEER and increased paracellular permeability, which can be reversed by tofacitinib. This was confirmed by a similar observation in human iPSC-derived colon organoids from patients with UC, where treatment with TNF-α, IFN-γ, and IL-1β caused mislocalization of ZO-1, which could be restored by tofacitinib.^67^ Similarly, preincubation of intestinal organoids derived from patients with CD with filgotinib restored ZO-1 protein levels under inflammatory conditions.^62^ Finally, in co-cultures of Caco-2BBe cells and THP-1 macrophages, tofacitinib normalized the barrier-sealing protein junctional adhesion molecule (JAM-A) but did not affect other junctional proteins.^51^

Summarizing, inflammatory JAK-STAT-mediated signals affect the expression of E-cadherin, CLDN2, and JAM-A levels and hamper the formation of functional TJss. Tofacitinib can reverse these effects and normalize E-cadherin and CLDN2 levels, resulting in increased TEER and reduced paracellular permeability, which together restore barrier capacity.

Goblet cells play a crucial role in separating the microbiota from the intestinal epithelium by producing mucins that form the mucus layer. Goblet cells are depleted in the upper part of the crypts in patients with IBD, especially in those with UC.^52^ This loss of mucus-producing cells could play a role in the pathogenesis of IBD. Aguilar et al.^14^ reported an increase in secretory cell gene expression in remitting patients with CD treated with upadacitinib. Moreover, tofacitinib treatment in experimental models of IBD reduced goblet cell depletion and colitis activity.^37^ Conversely, an in vitro study showed that treatment of colorectal HT-29 cells with tofacitinib did not affect the expression of goblet cell differentiation-related genes and mucin production.^63^ This lack of effect may, however, reflect the use of a colorectal cancer–derived cell line and the in vitro, non-IBD context rather than the absence of a true biological effect in vivo. Together, JAKis can promote the mucosal barrier through improved epithelial barrier function and potential preservation of mucus-secreting goblet cells, although, to date, no data are available on their effects on mucin expression.

#### Tofacitinib alters epithelial homeostasis and repair dynamics

The intestinal epithelium is the fastest self-renewing tissue in the human body, with a turnover rate of 3-5 days.^68^ A balance between proliferation, cell death, and migration is essential to maintain a healthy and intact intestinal epithelium, especially during inflammation. The effects of JAKis on intestinal stem cells remain largely unexplored. One study showed increased stem and secretory cell gene expression in the colonic tissue of patients with CD in remission after upadacitinib treatment.^14^ In contrast, another study demonstrated that tofacitinib reduced the proliferation of crypt cells in the jejunum of mice, while the numbers of goblet cells remained unchanged and those of Paneth cells increased.^69^ In line with a role for JAK-STAT signaling in epithelial regeneration, tofacitinib also blocked IFN-γ-induced stem cell expansion in mouse enteroids.^70^ Furthermore, in human HC and UC colonoids, tofacitinib treatment was associated with higher LGR5 protein levels compared with TNF+Poly(I:C) treatment alone, suggesting modulation of stemness.^71^

Apart from the effects on stem cells, JAK inhibition also influences epithelial repair and turnover. In a wound healing assay with Caco-2 cells, already low concentrations of tofacitinib resulted in impaired migration, defined by a nonconfluent cell layer compared with the control.^37^ These findings were corroborated in vivo by making small wounds in the colon of wild-type BL/6 mice and tracing wound healing over time. While low-dose tofacitinib did not affect wound closure, higher concentrations resulted in a 15% reduction in wound healing compared with the controls.^37^ Of note, these experiments were all performed without inducing inflammation. It is therefore conceivable that the effects of tofacitinib could not be detected because of the lack of JAK-STAT pathway activation. Regarding proliferation, tofacitinib did not alter the proliferation marker Ki67 in these mouse colonic tissues and organoids.^37^ One study addressed proliferation in the context of IBD and cancer in a chronic azoxymethane/DSS-colitis mouse model, where the difference between early and late tofacitinib administration was assessed (ie, after the first or second cycle of DSS). In disease-free control mice, Ki67 was only expressed in epithelial cells in the crypts, whereas in colitis-induced mice, there was more apical Ki67 expression.^42^ Thus, tofacitinib treatment significantly increased the percentage of Ki67^+^ cells, specifically in the colonocytes of HC and UC human colonoids.^71^ Taken together, tofacitinib impairs wound healing but also maintains or even increases proliferation.

The effects of tofactinib treatment on apoptosis appear more heterogeneous and inhibitor-specific. In oxazolone-induced colitis, tofacitinib treatment led to increased levels of epithelial cell death.^37^ This was supported by mouse colonic organoids, where tofacitinib did not alter the proliferation marker Ki67 but significantly increased caspase-3^+^ apoptotic cells.^37^ Moreover, in HC and UC colonoids, tofacitinib alone did not induce epithelial cell death, nor did it reduce TNF + Poly(I:C)-induced cell death.^71^ In contrast, upadacitinib treatment of HC and CD enteroids blocked IFN-γ-induced cytotoxicity, marked by decreased levels of cleaved caspase-3.^72^ Similarly, in HC organoids, tofacitinib increased cell viability following IFN-γ/TNF-α exposure.^57^ Consistent with these findings, filgotinib restored viability in IFN-γ/TNF-α-treated CD organoids, which is comparable to that achieved with upadacitinib and tofacitinib. However, in HT-29 cells, upadacitinib and ruxolitinib reversed IFN-γ/TNF-α-induced cytotoxicity, whereas filgotinib exerted only modest effects on viability.^73^

Overall, while tofacitinib modulates stemness, maintains proliferation, and impairs wound healing at higher doses, its effects on apoptosis are context dependent and could be detrimental to epithelial repair during inflammation. Notably, these effects differ from those of more selective JAKis since they do inhibit apoptosis in different organoid models. Future studies should investigate the effect of selective JAKis on epithelial homeostasis and repair.

#### Tofacitinib dampens pro-inflammatory cytokine production in the intestinal epithelium

Immune factors such as cytokines play a pivotal role in maintaining epithelial homeostasis, and multiple cytokines are increased in IBD (ie, TNF-α, IFN-γ, and IL-1β). Ruxolitinib has been shown to dose dependently inhibit IL-13-induced CCL26 secretion in Caco-2 cells,^58^ although the relevance of this finding for IBD remains unclear. Ruxolitinib also reduces *TNF* and *IFNG* transcription in HCT116 cells, and *TNF* and *IL6* transcription in Caco-2 cells stimulated with LPS, TNF-α, IFN-γ, and IL-1β.^56^ Similar anti-inflammatory effects were observed with tofacitinib. In intestinal organoid monolayers obtained from patients with UC, but not from HCs, tofacitinib inhibited *TNF* transcription, suggesting disease-context specificity.^20^ In addition, tofacitinib reduced IL-1β and IL-18 secretion in IFN-γ/TNF-α-stimulated HC organoids.^57^ Comparable anti-inflammatory effects were observed in HT-29 cells, where tofacitinib decreased both IL-6-induced TNF-α, IL-6, and IL-10 levels and *Escherichia coli*–induced IL-1β and IL-18 levels.^50^ Mechanistically, these effects may partly relate to the suppression of TNF-α-induced NF-κB signaling, as tofacitinib reduces NF-κB nuclear translocation^50^ and reverses TNF-α-induced *NFKB1* transcription in Caco-2 cells.^65^ These findings suggest that JAK inhibition in intestinal epithelial cells consistently attenuates pro-inflammatory cytokine signaling and transcriptional responses, although the extent and context dependency of these effects remain to be fully defined in IBD.

IL-8 secretion by the epithelium has also been linked to IBD,^74^ and like other cytokines, levels of *IL8* mRNA and cytokine secretion were reduced on tofacitinib treatment of both cytokine-stimulated UC and HC epithelial monolayers and C2BBe1 colorectal cells.^20^^,^^75^ However, apical IL-8 secretion by Caco-2 cells during co-culture with stimulated THP-1 macrophages was unaffected by tofacitinib, whereas IL-1β secretion was reduced.^21^ Overall, there is substantial evidence that tofacitinib decreases multiple pro-inflammatory cytokines in the intestinal epithelium (eg, TNF-α, IFN-γ, IL-6, IL-10, IL-1β, IL-18, and IL-8) (Figure 3), although these findings are derived from heterogeneous experimental settings and inflammatory stimuli.

These studies indicate that tofacitinib broadly modulates intestinal epithelial homeostasis by dampening pro-inflammatory cytokine signaling, improving barrier integrity, and altering proliferation and repair processes in a highly context-dependent manner. However, data on selective JAKis remain limited, underscoring the need for further studies to clarify their effects on epithelial function and mucosal healing in IBD.

## Discussion and Future Perspectives

In this review, we aimed to map the current evidence on the effects of JAKis on the main cell types involved in IBD to better understand mechanisms contributing to treatment (non-)response. Although the heterogeneity of the included literature enriches the scope of this review, it also introduces interpretative challenges. Variability in experimental design, cellular sources, costimulatory signals, and JAKi characteristics, including type, administration timing, concentration, and exposure duration, introduces considerable heterogeneity, posing challenges for cross-study comparisons.

### Translating Mechanistic Insights to Clinical Efficacy of JAKis

Despite limitations in the current literature, valuable observations have emerged that align with previous clinical findings. For instance, clinical trials have shown currently approved JAKis to be more effective in UC than in CD, with only upadacitinib achieving stringent clinical endpoints in trials. In line with these findings, most available data on UC and CD, primarily involving peripheral monocytes, indicate a higher sensitivity in UC than in CD to JAKi-induced reduction of pro-inflammatory cytokine production and modulation of inflammation-related membrane receptor expression.^10^^,^^11^^,^^27^ This could partly be due to differential underlying pathogenic inflammatory mechanisms, involving diverse mucosal cell types. Both myeloid and lymphoid cell types display differential responses to JAK-STAT inhibition. In general, JAK inhibition has an anti-inflammatory effect on T cells, with CD8^+^ cells possibly being more affected compared to CD4^+^ cells. A similar anti-inflammatory pattern is observed in epithelial cells. By contrast, myeloid cells exhibit both anti-inflammatory and pro-inflammatory responses to JAK inhibition, suggesting a context-dependent effect influenced by differentiation state, microenvironment, and costimulatory cues.^18^^,^^19^^,^^28^^,^^38^ Microbial ligands such as LPS and NOD2 ligands warrant investigation in the context of paradoxical effects of JAK inhibition on myeloid cells. A recent study on entheseal myeloid cells suggests a similar counterproductive mechanism, possibly mediated via disruption of IL-10 signaling.^76^ Nonetheless, the overall anti-inflammatory effects, partly driven by lymphoid and epithelial cells, are sufficient to ensure therapeutic efficacy in a subset of patients.

Moreover, compelling evidence shows that the effects of JAKis on immune cells and epithelium are dose dependent.^12^^,^^13^^,^^27^ These findings indicate a need for personalized dosing strategies. Potential off-target effects of high-dose JAKis remain poorly characterized but may offer an additional rationale to avoid dose escalation, as does the observed increase in coagulation-inducing tissue factor in light of thrombosis-related warnings issued by the European Medicines Agency and Food and Drug Administration.^77^^,^^78^ Given the lack of comparative studies, no definitive conclusions can be drawn regarding differential effects on cell types based on JAKi selectivity, nor on the effects of dose escalation on cellular mechanisms, although such differences may exist. Of note, in vitro administration likely does not reflect the physiological conditions in patients with IBD, who typically take medication once or twice daily and often have chronic inflammation established long before initiating JAKi therapy. Most in vitro studies use JAKi concentrations (100-2000 nM) that exceed tofacitinib serum levels (∼69 nM) but fall within colonic tissue concentrations measured in treated patients with UC (∼1700-3400 nM), although some epithelial assays use supraphysiological doses.^79^ Higher mucosal tofacitinib concentrations were shown to correlate with response, while serum levels did not, underscoring the importance of local tissue exposure.^79^ Beyond systemic pharmacokinetics, intracellular drug availability may be further modulated by membrane transporters that actively regulate JAKi uptake and efflux. In UC-derived organoids, tofacitinib uptake was dependent on multidrug and toxin extrusion protein 1 levels.^80^ Additionally, equilibrative nucleoside transporters have been implicated in tofacitinib uptake.^20^ By contrast, filgotinib was identified as a P-glycoprotein substrate, although clinically relevant effects of P-gp modulation on drug exposure appeared limited in HCs.^81^ IBD is further associated with increased intestinal P-gp expression, particularly in patients failing medical therapy, suggesting a role for efflux transporter upregulation in treatment resistance.^82^ Notably, P-gp expression has also been described in pro-inflammatory Th17 cells, where it contributes to reduced sensitivity to glucocorticoids, supporting a role for ABC transporters in immune cell intrinsic drug resistance mechanisms.^83^ Moreover, several polymorphisms in transporter genes, particularly within *ABCB1*, have been associated with altered transporter expression and function in IBD, including variants, such as C3435T (extensively reviewed by Wolf et al.^84^), which have been linked to UC susceptibility and altered epithelial barrier homeostasis.^85^^,^^86^ These findings suggest that variability in transporter expression and activity across patients, driven by inflammation, cellular heterogeneity, and potentially genetic variation, may contribute to interindividual variability in intracellular JAKi exposure and therapeutic responsiveness.

### Future Outlook

By synthesizing the currently available cell-specific evidence on intestinal JAK inhibition, this review reveals substantial gaps in the literature. Most existing evidence focuses on mucosal compartments, while several relevant cell types, including B cells, dendritic cells, goblet cells, epithelial stem cells, fibroblasts, and CD8^+^ T cells, remain understudied. Fibroblasts are of particular interest, given the potential relevance of JAK-STAT signaling in intestinal fibrosis and fistulizing CD, settings in which JAKis have emerged as candidate therapies, yet stromal mechanisms remain poorly defined.^87^, ^88^, ^89^

These gaps are best addressed through longitudinal human mucosal sampling. Paired biopsies obtained before and after JAKi treatment, preferably from the same intestinal segment and linked to clinical, endoscopic, and histologic responses, would provide the most direct insight into in vivo drug effects. Sampling should also account for anatomical location, as drug effects may differ between ileal and colonic disease because of regional differences in epithelial biology, immune composition, stromal remodeling, microbiota exposure, and baseline cytokine signaling.^31^^,^^90^ Ex vivo perturbation models and organ-on-a-chip systems should complement, rather than replace, these longitudinal tissue studies. Baseline patient-derived tissue can be exposed to different JAKis under defined inflammatory conditions, using stimuli such as LPS, NOD2 ligands, TNF-α, or IFN-γ, after which cellular responses can be benchmarked against post-treatment mucosal tissue from the same patient. If these ex vivo responses accurately emulate in vivo changes, such models could provide a patient-specific platform for comparing inhibitor-specific effects, dose-response relationships, and cell-type-specific sensitivity before or alongside clinical treatment.

Future studies should also account for pharmacologic variables that are often simplified in experimental systems. JAKis differ in relative JAK isoform selectivity, but this selectivity is dose-, tissue-, assay-, and cell-context-dependent rather than absolute. At higher exposures, individual agents may inhibit broader JAK-dependent cytokine pathways, and some also show compound-specific off-target binding to non-JAK kinases.^91^ We therefore require better characterization of drug concentrations in blood and intestinal tissue and relevant metabolites such as the active metabolite of filgotinib. These measurements could help align experimental dosing with clinically relevant exposure and clarify whether observed cellular effects reflect intended JAK-STAT pathway inhibition, broader kinase activity, or indirect consequences of reduced inflammation.

Finally, integrating spatial and multimodal profiling into stronger translational study designs can move the field from descriptive cellular associations toward spatially resolved mechanisms of JAK inhibition. Paired sampling, comparator-treated cohorts, and pharmacologic exposure data can help distinguish direct drug effects from baseline disease heterogeneity and secondary changes accompanying inflammatory resolution. Spatial transcriptomics platforms such as CosMx and Xenium can map treatment-associated gene expression changes to defined mucosal niches, where spatial proteomics can localize pathway activity and immune-cell-type phenotypes associated with treatment response.^92^^,^^93^ These approaches can help define where JAKis act, which cellular neighborhoods are altered, and how local pathway inhibition translates into mucosal repair.

## Declaration of Generative AI and AI-Assisted Technologies in the Writing Process

During the preparation of this work, the authors used ChatGPT-5.3 (OpenAI) solely to improve linguistic clarity and legibility. After using this tool, the authors thoroughly reviewed and edited all AI-generated text as needed and take full responsibility for the content of the publication.
